# The Potential of A Disintegrin and Metalloproteinase (ADAM) Proteins as Clinically Relevant Biomarkers in Colorectal Cancer: A Comprehensive Analysis

**DOI:** 10.3390/cancers18071127

**Published:** 2026-04-01

**Authors:** Adrianna Romanowicz, Marta Łukaszewicz-Zając, Barbara Mroczko

**Affiliations:** 1 Department of Neurodegeneration Diagnostics, Medical University of Bialystok, 15-276 Bialystok, Poland; mroczko@umb.edu.pl; 2 Department of Biochemical Diagnostics, Medical University of Bialystok, University Clinical Hospital in Bialystok, 15-276 Bialystok, Poland; marta.lukaszewicz-zajac@umb.edu.pl

**Keywords:** colorectal cancer, disintegrin and metalloproteinase, biomarkers

## Abstract

Colorectal cancer (CRC) remains a major global health challenge, primarily due to late-stage diagnosis and high metastatic potential. Effective management requires novel diagnostic and prognostic strategies, with a growing focus on molecular biomarkers. A Disintegrin and Metalloproteinase (ADAM) proteins, characterized by unique proteolytic activity, play a fundamental role in tumorigenesis by regulating tumor growth, epithelial–mesenchymal transition (EMT), and metastasis. Based on recent investigations, among all ADAMs, ADAM8, ADAM9, ADAM12, ADAM15, and ADAM17 have been proved to play an important role in the CRC pathogenesis. Thus, this review underscores the potential of selected ADAM family members as promising candidates for biomarkers of CRC. Elevated ADAM8, ADAM9, ADAM12 and ADAM17 levels were observed in CRC tissues and correlated with more advanced tumor stage, while increased serum ADAM15 concentrations associated with the presence distant metastases. Moreover, ADAM9, ADAM12, ADAM15 and ADAM17 levels were associated with poorer survival, whereas ADAM8 overexpression was found to be independent prognostic factor for CRC patients’ survival. In addition, the measurement of serum ADAM15 concentrations, especially in combination with well-established tumor marker–CEA improved the diagnosis of patients with this malignancy. In conclusion, selected ADAM are critical contributors to the development and progression of CRC, affecting tumor growth, EMT, and metastasis. ADAM8, ADAM9, ADAM12, ADAM15 and ADAM17 were identified as promising biomarkers for the assessment of CRC progression and proved to be prognostic indicators for patients’ survival. Further validation through large prospective studies and standardized assays is necessary to establish their potential in clinical practice.

## Simple Summary

Colorectal cancer (CRC) is often diagnosed at advanced stages, which contributes to poor outcomes and high mortality, highlighting the need for novel biomarkers. This review focuses on A Disintegrin and Metalloproteinase (ADAM) family proteins, particularly ADAM8, ADAM9, ADAM12, ADAM15, and ADAM17, which are involved in tumor growth, epithelial–mesenchymal transition, and metastasis. These proteins are frequently elevated in CRC tissues or serum and are associated with more advanced disease, distant metastases, and reduced patient survival. Overall, selected ADAM proteins show strong potential as diagnostic and prognostic biomarkers in CRC, although further large-scale clinical studies are needed to confirm their clinical utility.

## 1. General Overview of Colorectal Cancer

Colorectal cancer (CRC) is among the most frequently diagnosed cancers worldwide, currently ranking third in terms of global incidence and second in cancer-related deaths [1,2]. According to recent data from the International Agency for Research on Cancer, approximately 1.9 million new CRC cases and around 904,000 related deaths were recorded in 2022, resulting in an estimated mortality rate of about 20% [2,3]. CRC develops through a multistep process of carcinogenesis that typically begins with precancerous lesions, such as adenomatous polyps. Depending on the genetic background, CRC can be categorized into sporadic, hereditary, or familial forms [4]. Its development is driven by a complex interaction between inherited susceptibility and environmental influences. It is estimated that roughly three-quarters of CRC cases result from the combined effects of genetic predisposition and environmental exposures [5,6]. While hereditary CRC represents a smaller proportion of all cases, they hold significant clinical importance. Mutations in oncogenes and in tumor suppressor genes, as well as in deoxyribonucleic acid (DNA) repair genes, can initiate and promote colorectal tumorigenesis [5,6]. Hereditary factors are estimated to account for around 20% of CRC cases, classified under familial CRC [7,8].

Given the substantial global burden of CRC, the development of effective diagnostic methods for early detection remains a top priority. The prognosis of CRC strongly depends on the stage at diagnosis. Patients diagnosed with CRC may experience symptoms such as rectal bleeding, changes in bowel habits, abdominal pain, persistent fatigue, anemia, or unintended weight loss. The diagnosis of CRC involves a comprehensive approach that integrates laboratory tests with imaging techniques. Among the tumor markers investigated, carcinoembryonic antigen (CEA) remains the most extensively analyzed; however, its limited sensitivity in early cancer stages restricts its application in screening programs. Other potential biomarkers, including cancer antigen 19-9 (CA 19-9), cancer antigen 50 (CA 50), and cancer antigen 72.4 (CA 72.4), have also been explored, but none have proven sufficiently accurate for early diagnosis of this malignancy [9,10]. Despite extensive research efforts over recent years, a universally accepted biomarker for the early detection of CRC has yet to be established. The limited correlation of biomarkers with patient stratification and the overall precision oncology framework poses a significant medical challenge in clinical practice. Molecular testing is recommended; however, there are considerable disruptions between the discovery and validation of biomarkers and their routine clinical application. The primary limitations within precision oncology frameworks include inadequate predictive power (as biomarkers tend to be more prognostic than predictive), tumor heterogeneity, and the gaps that arise during biopsy procedures, test ordering, reporting, and interpretation. Thus, the critical challenges are focused on significant barriers to effective integration, including high costs and time constraints, as well as a lack of training in interpreting complex genomic data. Strategies for enhanced connectivity involve the implementation of automatic reflex testing, where molecular testing is initiated by pathology upon diagnosis, which has shown a reduction in delays and an increase in testing rates. Additionally, transitioning to AI-driven analysis of multi-omics data, encompassing proteomics and genomics, can enhance prediction accuracy compared to single-gene biomarkers. The application of blood-based monitoring, or liquid biopsy, to detect circulating tumor DNA can mitigate tissue insufficiency and enable real-time monitoring of clonal evolution. The formation of specialized boards to review complex cases ensures that molecular profiling is effectively translated into actionable treatment plans [11]. This underscores the pressing need to develop noninvasive, reliable diagnostic tools, such as blood-based biomarkers, to facilitate early diagnosis and improve clinical outcomes.

CRC screening strategies are divided into two main types: population-based and opportunistic [12]. Population-based programs, coordinated by public health authorities, aim to detect early or asymptomatic cases. However, they are often expensive and have low participation rates. Opportunistic screening, performed during routine medical visits, is more affordable and has higher patient compliance, although it often misses asymptomatic individuals. Early screening is recommended for people at increased risk, including those with a family history of CRC, microsatellite instability, DNA mismatch repair deficiencies, or hereditary mutations. For example, individuals with Lynch syndrome are advised to begin screening between ages 20 and 25, or 2–10 years earlier than the youngest affected family member, while those with familial adenomatous polyposis (FAP) should start colonoscopy screening between ages 10 and 18 [12]. In this context, there is increasing interest in identifying novel regulators involved in CRC progression and tumor microenvironment (TME) interactions. Among these, members of the A Disintegrin and Metalloproteinase (ADAM) family have emerged as important mediators of cell signaling, proteolysis, and cellular crosstalk. Therefore, this review focuses on selected ADAM proteins as promising candidates for biomarkers in CRC, with particular emphasis on their structural and functional characteristics

## 2. Methodology of Literature Search

A systematic literature search was conducted to identify publications related to the role of ADAM proteins in CRC, with particular emphasis on their diagnostic and prognostic potential. The search was performed using the electronic database PubMed. The literature search was initiated using a combination of keywords and search terms, including “ADAM”, “ADAM metalloproteinases”, “colorectal cancer”, “CRC”, “biomarkers”, “prognosis”, and “diagnosis”. An initial broad query (“ADAM in colorectal cancer”) yielded 236 results. Titles and abstracts were subsequently screened to identify potentially relevant studies. Inclusion criteria comprised studies investigating the expression, as well as concentrations (tissue and circulating levels), function, or clinical significance of ADAM family proteins in CRC, including both experimental and clinical research. Exclusion criteria included studies not related to CRC, studies focusing on other ADAM-independent mechanisms, and articles lacking sufficient experimental or clinical data.

To facilitate full-text evaluation, filters for article availability were applied, including “Abstract”, “Free full text”, and “Full text”. Additionally, a publication date filter (2010–2026) was introduced to improve the relevance and timeliness of the selected studies, which reduced the number of records to 212. Following this initial screening, full-text articles were assessed for eligibility. Based on a critical evaluation of the literature, ADAM8, ADAM9, ADAM12, ADAM15, and ADAM17 were selected as key ADAM family members for detailed analysis. The final set of publications (81) comprised both original research articles and review papers. However, particular emphasis was placed on original studies (approximately 60%), as they provided primary experimental and clinical evidence supporting the biomarker potential of ADAM proteins in CRC. Notably, the majority of these studies were published between 2020 and 2025, reflecting the growing interest in ADAM proteins in the context of CRC.

## 3. Structural Organization of ADAM Proteins

**Figure 1:**
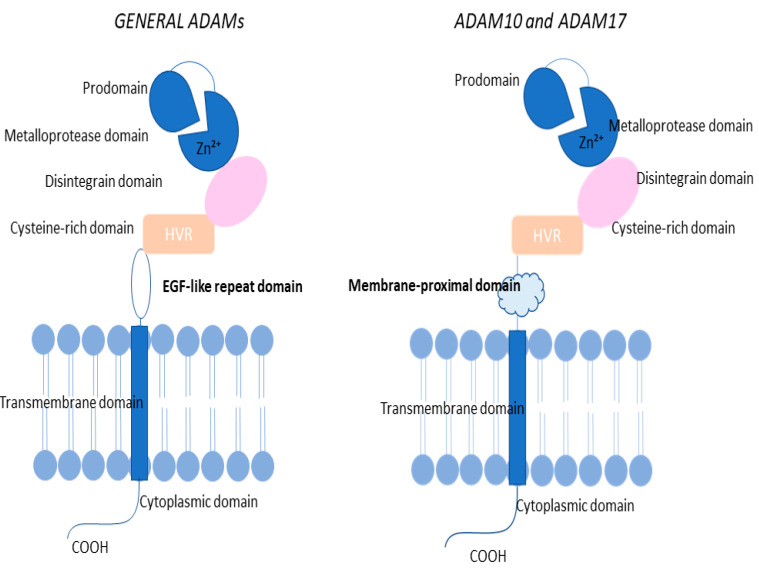
Structural characteristics of general ADAMs, ADAM10, and ADAM17.

ADAM proteins share a conserved multi-domain architecture. Their extracellular portion contains a prodomain with two convertase recognition sites, a Zn^2+^-dependent metalloprotease domain (MP), a disintegrin region, and a cysteine-rich segment that includes a hypervariable region (HVR) [13]. This is followed by an epidermal growth factor (EGF)-like repeat, a transmembrane span, and a cytoplasmic tail at the C-terminus [13,14,15,16,17,18,19,20,21,22,23,24,25,26,27] (Figure 1). ADAM10 and ADAM17 deviate from this canonical architecture. In these two proteins, the cysteine-rich domain together with the EGF-like domain is replaced by a membrane-proximal domain [14,15].

Structurally, the prodomain directly precedes the MP domain, and its interaction with this region is pivotal for activating the proteolytic function of ADAM proteins. It acts as a molecular chaperone and suppresses the activity of the zymogen state. Together with the signal sequence (SS), it also directs the intracellular trafficking of the immature protein [16]. The MP domain itself carries the conserved HEXGH motif shared with matrix metalloproteinases (MMPs), which underlies its catalytic capacity. However, in certain ADAM family members, such as ADAM11, ADAM22, and ADAM23, alterations within this motif render them non-proteolytic, shifting their primary roles toward regulating cell–cell and cell–matrix adhesion rather than enzymatic activity [17,18]. Extending toward the C-terminal end from the MP domain is the disintegrin (D) region, which can bind integrins and mediate both cell–cell and cell–matrix interactions [19]. Adjacent to this is the cysteine-rich (C) segment, which in ADAM10 forms a continuous structural unit with the D domain [24,25]. The C region plays a key role in regulating protease activity as well as in recognizing and binding substrates, and it also influences how the D domain interacts with integrins [20]. Beyond this point, most ADAMs (except for ADAM10 and ADAM17) possess a 30–40-amino-acid EGF-like motif, whose precise function remains unclear. The transmembrane (TM) sequence stabilizes the adjacent, intrinsically disordered cytoplasmic region. In ADAM10, it may promote homodimer formation within the membrane [27]. The TM domain also likely supports other protein and lipid interactions through association with specific lipid microdomains [21,22]. The cytoplasmic or C-terminal tail of ADAM proteins contains an SH3 (Src homology 3)-binding motif and shows considerable variability in both length and sequence [23]. Its phosphorylation can influence shedding activity, as seen with threonine phosphorylation in ADAM17 [26]. Strong SH3-binding capability is not universal across ADAMs. A phage display study showed that only ADAM8, ADAM9, and ADAM15 efficiently recovered SRC homology 3-binding (SH3-binding) phages, indicating selective high-affinity interactions. Notably, the cytoplasmic domain is dispensable for shedding in ADAM10 and ADAM17, suggesting the presence of other regulatory mechanisms [28]. The structural organization of ADAM proteins determines their proteolytic activity, particularly their ability to mediate ectodomain shedding of membrane-bound proteins. This process regulates the availability of growth factors and adhesion molecules, thereby influencing key cellular interactions and signaling events relevant to CRC progression.

## 4. Mechanisms of ADAM Metalloprotease Regulation

### 4.1. Prodomain Cleavage and Activation

The majority of ADAM proteins are synthesized in an inactive zymogen form and require prodomain removal or displacement to achieve catalytic activity. This activation usually occurs along the secretory pathway and is driven by proprotein convertases, including furin, that cleave a conserved RX(R/K)R–Arginine (R)/any amino acid (X)/arginine or lysine (R/K)/arginine (R)-consensus sequence [29,30]. The prodomain acts as a so-called “cysteine switch”, a regulatory mechanism also described for MMPs. In this process, a conserved cysteine residue interacts with the zinc ion located in the catalytic domain, thereby preventing access to the active site. Enzymatic activation occurs only after this interaction is disrupted through proteolytic cleavage, reduction, or allosteric changes [31,32]. In addition to prodomain-dependent regulation, ADAM protease activity is further controlled by the structural organization and conformational changes in its extracellular domains. Structural analyses of ADAM10 indicate that intramolecular interactions promote an autoinhibited conformation in which the cysteine-rich region associates with the MP, thereby restricting substrate access. Within this configuration, the D and C domains adopt an elongated and structurally stable arrangement. This structure is maintained by disulfide bonds and hydrophobic interactions, which preserve structural integrity and maintain catalytic inactivity [24,32].

**Figure 2:**
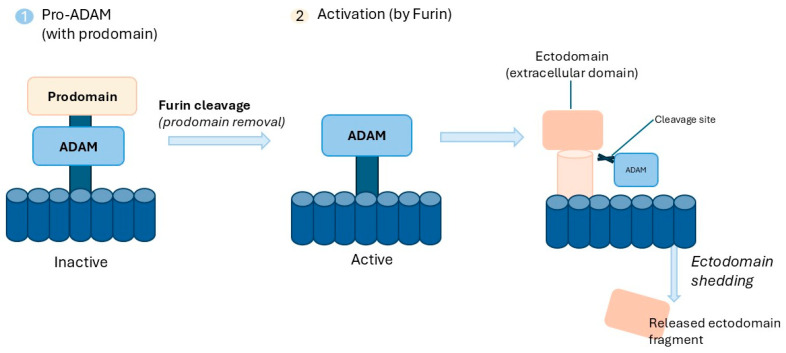
Mechanism of ADAM activation and ectodomain shedding.

Conformational activation of ADAM proteases may also be driven by redox-dependent rearrangements of disulfide bonds within conserved thioredoxin-like Cys-X-X-Cys (CXXC) motifs, which contribute to exposure of the catalytic site. These disulfide exchanges are mediated by protein disulfide isomerase (PDI) and promote reorientation of extracellular domains, thereby regulating proteolytic shedding activity [25,33,34,35]. In addition, PDI has been reported to bind directly to the membrane-proximal domain (MPD) of ADAM17, promoting reorientation of the extracellular domains and regulating its shedding activity [36]. Figure 2 illustrates the mechanism of ADAM activation and ectodomain shedding [24,25,29,30,31,32,33,34,35,36].

### 4.2. Accessory Protein–Mediated Trafficking, Localization, and Maturation

The regulation of ADAM proteins is closely linked to their trafficking, localization, and maturation processes, which rely on specific accessory proteins, most notably tetraspanins (Tspans) and rhomboid-like proteins (iRhoms). Tspans represent a highly conserved family of proteins, comprising 33 members in mammals, each featuring four TM domains, cytoplasmic N- and C-terminal region, as well as two extracellular and one intracellular loop. These proteins interact with specific partner molecules and are capable of forming tetraspanin-enriched microdomains through interactions between Tspans molecules themselves [37]. ADAM10-interacting Tspans display varying degrees of amino acid sequence similarity, with closely related members such as Tspan5 and Tspan17 and more divergent proteins, including Tspan10 and Tspan15. These proteins were classified as the TspanC8 subgroup due to the presence of eight cysteine residues [38]. In cell-based systems, elevated expression of any of the TspanC8 family members facilitates the release of ADAM10 from the endoplasmic reticulum (ER) and supports its proteolytic maturation [37]. Additionally, distinct TspanC8 proteins show different subcellular distributions when expressed in cell lines. Some direct ADAM10 to intracellular compartments, while others promote its transport to the plasma membrane. Increasing evidence also indicates that some TspanC8 members differentially regulate ADAM10-dependent shedding of specific substrates. For instance, Tspan5, Tspan10, and Tspan14 have been shown to enhance Notch signaling, whereas Tspan15 and Tspan33 exert inhibitory effects. Similar substrate-selective regulation has been described for other ADAM10 targets: Tspan15 preferentially enhances N-cadherin proteolysis, whereas Tspan14 uniquely limits ectodomain shedding of the platelet collagen receptor (GP)VI [37,39].

iRhoms are a subgroup of rhomboid family proteins that share high sequence similarity to other rhomboid members but lack the key catalytic motif characteristic of active rhomboid proteases [40,41]. Consequently, iRhom1 and iRhom2 are considered inactive rhomboid proteins that lost their proteolytic activity during evolution while retaining important non-proteolytic functions, including roles in the regulation of the EGF and tumor necrosis factor-alpha (TNF-α) signaling pathways [42,43]. iRhom1 and iRhom2 play a pivotal role in ADAM17 function, overseeing both its maturation and trafficking to the cell membrane [44]. Additionally, iRhom2 is strongly expressed in immune cells during inflammation and is essential for ADAM17-mediated shedding of substrates such as TNF-α. In the absence of iRhom2, ADAM17 is retained in the ER, preventing substrate cleavage and leading to impaired inflammatory responses [45]. Some studies indicate that iRhom2 interacts with ADAM17 via both extracellular and TM domains. Binding to iRhom2’s first transmembrane domain (TMD1) is essential for ADAM17 trafficking from the ER to the Golgi and for its maturation, as disruption of this interaction blocks processing, mimicking iRhom2 loss. In addition, contacts involve a unique loop in iRhom2’s extracellular domain, located between TMD1 and TMD2, which engages three distinct sites in ADAM17’s extracellular domain. The most functionally significant of these is an unexpected interaction with the prodomain, proposed to keep it transiently associated with iRhom2 after furin cleavage, preventing premature activation until cytoplasmic signals trigger its release and enable full protease activity [46]. Moreover, the N-terminal region of iRhom2 contains key regulatory elements, including phosphorylation sites and binding motifs for the adapter protein FRMD8 (FERM Domain-Containing Protein 8), also known as iTAP (iRhom tail-associated protein), which stabilizes the iRhom2–ADAM17 complex. Pathogenic N-terminal mutations, such as those identified in oesophageal cancer, are associated with increased ADAM17 expression and enhanced epidermal growth factor receptor (EGFR) signaling, underscoring the functional and clinical relevance of this regulatory region [47,48]

### 4.3. Inhibition of ADAM Metalloproteases by TIMPs

The TIMP (tissue inhibitor of metalloproteinase) family (TIMPs 1–4) consists of natural inhibitors of ADAM metalloproteases [49]. Structural crystallography studies demonstrated that the N-terminal domain of human TIMPs associates with metalloproteinase catalytic domains at a 1:1 stoichiometry, suppressing enzymatic activity by interfering with Zn^2+^ coordination in the active site [50,51]. TIMP3 functions as a potent endogenous inhibitor of both ADAM17 and ADAM10, whereas TIMP1 displays comparatively weaker inhibitory activity toward ADAM10 [52,53]. Beyond general inhibition, TIMPs also regulate substrate-specific shedding events. For instance, recombinant TIMP3 strongly inhibits resting and activated platelet ADAM10 activity, whereas exogenous TIMP2 exerts only modest inhibitory effects on ligand-induced GPVI shedding [53]. Activation of Extracellular Signal-Regulated Kinase (ERK) or p38 Mitogen-Activated Protein Kinase (p38 MAPK) signaling promotes a conformational transition of ADAM17 toward a monomeric state, facilitating the dissociation of TIMP3 [26,54]. In particular, phosphorylation of ADAM17 at threonine 735 (Thr735) by p38α MAPK enhances its proteolytic capacity, while simultaneously diminishing TIMP3 binding [26,54]. Similarly to ADAM17, ADAM10 is capable of forming dimers in a manner dependent on its TM domain, and it may also undergo phosphorylation within its intracellular region [27,55]. Notably, transient ADAM17 activation has been reported to occur independently of prodomain removal or TIMP3 release, implying the existence of highly dynamic regulatory mechanisms operating at the catalytic site [56]. Moreover, the cytoplasmic tails of ADAM10 and ADAM17 appear to be dispensable for enzymatic activity, and instead may exert inhibitory effects, for example, by promoting ER retention or through interactions with regulatory partner proteins [56,57,58]. Altogether, these regulatory mechanisms determine ADAM protease activity and may influence their role in CRC pathogenesis by modulating signaling pathways and proteolytic processes relevant to tumor progression.

## 5. ADAM Metalloproteinases in Colorectal Cancer

The biological functions of ADAM proteins, particularly their role in ectodomain shedding and modulation of signaling pathways, have important clinical implications in CRC. Alterations of these processes may lead to changes in protein expression in tumor tissue and the release of soluble molecules into the serum, which can be detected and may serve as potential biomarkers. Consistent with this, dysregulation of the ADAM family has been associated with various pathological conditions, including cancer. In CRC, ADAM proteins actively contribute to disease progression through their involvement in cell–cell communication and proteolytic processing. Overexpression of selected ADAMs (such as ADAM8, ADAM9, ADAM12, ADAM15, and ADAM17) has been linked to enhanced tumor progression, including increased proliferation, invasion, and tumor growth [58,59,60,61,62,63,64,65,66,67,68,69,70,71,72,73,74,75,76,77,78]. Table 1 provides a comparison of selected ADAMs in CRC.

### 5.1. ADAM8 in CRC: From Expression Patterns to Biomarker Potential

ADAM8 is a transmembrane glycoprotein of the ADAM family that contains metalloproteinase and disintegrin domains. It exhibits proteolytic activity and participates in multiple biological processes, including cell adhesion, cell fusion, and intracellular signaling. Moreover, ADAM8 is involved in the ectodomain shedding of membrane-associated proteins, and it has been linked to inflammatory responses. Mechanistically, ADAM8 promotes tumorigenesis by enhancing angiogenesis, increasing invasive and migratory capacities of cancer cells, and inhibiting apoptosis [59,60,61,62]. Elevated ADAM8 expression has been reported more frequently in CRC tissues than in adjacent non-tumorous samples [62]. Experimental studies using CRC cell lines have demonstrated that reduced ADAM8 activity is associated with decreased cell growth and proliferation, accompanied by increased apoptotic responses. Additionally, multivariate analyses indicate that ADAM8 may serve as an independent negative prognostic factor for both overall survival (OS) and disease-free survival (DFS) (both *p* < 0.001). Notably, 5-year DFS rates were significantly worse in patients with ADAM8-positive tumors compared with ADAM8-negative cases, particularly in subgroups including T3–T4 stage, N0 stage, Tumor–Node–Metastasis (TNM) stage II, adenocarcinoma histology, moderately differentiated tumors, and male patients (*p* < 0.05) [63]. Other investigators reported that increased ADAM8 expression in CRC tissue is significantly associated with clinicopathological features, including advanced stage according to the American Joint Committee on Cancer (AJCC), greater depth of tumor invasion, lymph node involvement, and the presence of distant metastases (*p* < 0.05) [63]. Furthermore, ADAM8 has been shown to decrease the expression of epithelial markers, such as E-cadherin, while increasing mesenchymal markers, including vimentin and N-cadherin, suggesting a role in promoting epithelial–mesenchymal transition (EMT) in CRC cells. In addition, ADAM8 appears to enhance the invasive capacity of these cells and to regulate EMT through the TGF-β/Smad2/3 signaling pathway [63]. Taken together, despite the substantial body of evidence supporting the role of ADAM8 in CRC progression, several limitations should be considered. This study is based on tissue-derived analyses, including immunohistochemical (IHC) staining and gene expression assays, without parallel evaluation of the concentration of ADAM8 in serum, which limits its applicability as a non-invasive biomarker. Although the survival analysis was performed on a relatively large cohort of 342 patients, molecular analyses were conducted on a limited number of tissue samples (30), and no independent validation cohort was included [63].

### 5.2. ADAM9 in CRC: From Expression Patterns to Biomarker Potential

ADAM9 is a membrane-bound metalloproteinase that is significantly upregulated in several human carcinomas, including CRC. Consistent with this observation, IHC revealed increased ADAM9 expression in CRC tissues in comparison to non-tumor samples, which was further supported by molecular analyses showing elevated messenger ribonucleic acid (mRNA) levels in tumor specimens compared to non-cancerous tissue. Upregulation of ADAM9 via transfection in HT-29 (American Type Culture Collection, Manassas, VA, USA) cells promoted cell invasion, while exerting no significant effect on proliferative activity [64]. Other investigations showed that the secreted ADAM9 isoform (ADAM9s) is markedly elevated in immature cancer-associated fibroblasts (CAFs) derived from CRC tissues. Silencing of ADAM9s in immature CAFs eliminated their pro-proliferative and pro-migratory effects on CRC cells. Additionally, CAF-derived ADAM9s have been associated with poorer survival in CRC patients with immature-type desmoplastic reaction (DR), likely through promotion of tumor cell proliferation and dissemination [65]. Ephrin-B signaling is involved in various physiological and pathological processes, including tumor metastasis. A study conducted by Chandrasekera et al. suggests that in mammals, ADAM9 cleaves ephrin-B1 and ephrin-B2 [66]. Knockdown of ADAM9 expression in SW620 (American Type Culture Collection, Manassas, VA, USA) and HCT116 (American Type Culture Collection, Manassas, VA, USA) cells suppresses migration and invasion, accompanied by reduced Akt (serine/threonine) kinase activity, as ephrin-B ligands antagonize Akt signaling. Akt serves as a central signaling node regulating multiple downstream pathways, including Wnt and mechanistic Target of Rapamycin (mTOR), both of which promote CRC cell migration and invasion. Notably, the downstream effects of ADAM9 knockdown differ between cell lines: Wnt signaling is reduced in SW620 (American Type Culture Collection, Manassas, VA, USA) cells with minimal impact on mTOR, whereas mTOR activity is suppressed in HCT116 (American Type Culture Collection, Manassas, VA, USA) cells. These findings indicate that ADAM9 promotes CRC cell migration and invasion through ephrin-B–dependent modulation of Akt, with context-dependent downstream signaling reflecting CRC heterogeneity [66]. Overall, current evidence on ADAM9 is predominantly based on in vitro and tissue-based studies, with no evaluation of serum levels and limited clinical validation. Furthermore, its effects appear to be model-dependent; for instance, the differing signaling responses in SW620 (American Type Culture Collection, Manassas, VA, USA) and HCT116 (American Type Culture Collection, Manassas, VA, USA) cells underscore the intrinsic heterogeneity of CRC and limit its reliability as a biomarker.

### 5.3. ADAM12 in CRC: From Expression Patterns to Biomarker Potential

ADAM12 expression was markedly elevated in human CRC tissues compared with corresponding normal mucosa, both at the mRNA and protein levels, as demonstrated in colonoscopic biopsy and surgical specimens [67]. Increased ADAM12 expression was significantly correlated with advanced tumor stage, greater depth of invasion, lymph node involvement, the presence of distant metastasis, and reduced OS in patients with CRC. Additionally, the mean apoptotic index (AI) was significantly lower in ADAM12-positive tumors than in ADAM12-negative tumors, further supporting its anti-apoptotic activity in vivo. Consistent with these observations, ADAM12 overexpression in CRC cells promoted proliferation and suppressed apoptosis by reducing caspase-specific activities. It also enhanced cell cycle progression through upregulation of cyclins and cyclin-dependent kinases (CDKs) and downregulation of CDK inhibitors. Silencing ADAM12 reversed these effects. Together, these findings indicate that ADAM12 contributes to tumor progression and survival in human CRC by promoting cell growth and limiting apoptotic processes [67]. Another study demonstrated that elevated ADAM12 expression was significantly associated with vascular invasion (*p* < 0.05), perineural invasion (*p* < 0.01), the presence of lymph node metastasis (*p* < 0.01), and advanced TNM stage (*p* < 0.001) [68]. Functional analyses confirmed that ADAM12 overexpression enhanced the proliferative and migratory capacity of CRC cells. This was accompanied by increased expression of EMT markers, including N-cadherin, Vimentin, and Twist, along with decreased E-cadherin expression (*p* < 0.01). Moreover, upregulation of ADAM12 was associated with elevated expression of proteins involved in the Wnt/β-catenin signaling pathway, such as β-catenin, phosphorylated GSK-3β (Glycogen Synthase Kinase-3 beta), c-MYC (cellular myelocytomatosis oncogene), and MMP-7 (*p* < 0.01). Importantly, treatment with the Wnt/β-catenin pathway inhibitor methyl 3-(4-methylphenylsulfonamido)benzoate (MSAB) attenuated the ADAM12-induced EMT process in CRC cells, indicating that ADAM12 exerts its effects at least partly through activation of this signaling pathway [68]. Additionally, a study by Hoorn et al. showed that high serum ADAM12 levels in patients from the CAIRO2 trial were associated with poorer OS, especially in metastatic rectal cancers and mesenchymal subtype tumors [69].

ADAM12 shows a consistent correlation with CRC progression across both tissue expression and serum levels. Its structural role in activating the Wnt/β-catenin pathway and EMT directly supports clinical observations of advanced tumor stage and reduced OS.

### 5.4. ADAM15 in CRC: From Expression Patterns to Biomarker Potential

Serum concentrations of ADAM15 and the well-known classic tumor markers for CRC, such as CEA and carbohydrate antigen 19-9 (CA19-9), were elevated in CRC patients compared to healthy controls. However, a statistically significant difference was observed only for CEA (*p* < 0.001). ADAM15 levels were significantly higher in CRC patients with distant metastases than in those without (*p* = 0.043). These findings suggest a significant role of ADAM15 in CRC pathogenesis and indicate its potential usefulness in predicting the occurrence of distant metastases [70]. Moreover, in our previous original paper, we evaluated whether circulating serum ADAM15 might be a candidate biomarker for CRC diagnosis, which was assessed by the calculation of diagnostic criteria. We investigated that the combined analysis of serum ADAM15 with a well-established tumor marker for CRC–CEA increased diagnostic sensitivity and may improve the diagnosis of patients with CRC [70]. Additional recent findings showed that ADAM15 expression is increased in both colon and rectal cancer tissues compared to adjacent normal mucosa. IHC analysis revealed varying levels of ADAM15 in tumor samples, with higher expression in primary tumors as compared to the normal intestinal mucosa, correlating significantly with decreased OS in CRC patients. These observations support the involvement of ADAM15 in CRC progression and its potential as a prognostic biomarker [71].

In contrast to ADAM12, the evidence for ADAM15 is more varied, but its tissue expression correlates with tumor progression; its serum concentration appears to be a more specific marker, showing significant elevation primarily in patients with distant metastases. This indicates that while both proteins (ADAM12 and ADAM15) contribute to CRC pathogenesis, ADAM12 provides a more integrated link between molecular mechanisms and clinical outcomes, whereas the diagnostic potential of ADAM15 in systemic circulation may be limited to specific metastatic subgroups.

### 5.5. ADAM17 in CRC: From Expression Patterns to Biomarker Potential

The protease ADAM17, also known as tumor necrosis factor-α-converting enzyme (TACE), is a type I transmembrane cell-surface metalloprotease [72]. Studies have consistently shown that ADAM17 is upregulated in both primary and metastatic CRC tissues compared with normal colonic mucosa or adjacent non-cancerous tissues [73,74]. Its immunoreactivity has been reported to be inversely correlated with transforming growth factor-α (TGFα) expression [73]. Increased ADAM17 expression was associated with metastasis and poorer prognosis in patients with CRC [74]. Moreover, ADAM17 knockdown decreased the expression of TGF-β/Smad pathway-related proteins, leading to reduced expression of migration- and invasion-associated proteins and consequent impairment of these cellular processes [73,75]. Furthermore, ADAM10, together with ADAM17, participates in the proteolytic shedding of the transmembrane protein Klotho (KL), a process additionally enhanced by insulin stimulation [75,76]. In a study by Chen et al., both mRNA and protein expression levels of testis-expressed *gene 14 (TEX14)* and ADAM17 were found to be significantly elevated in CRC tissues compared to non-tumor samples [77]. Patients characterized by poorer tumor differentiation, greater depth of invasion, and advanced TNM stage (III–IV) showed increased positivity rates for *TEX14* and ADAM17 expression. Increased expression of both proteins was also more frequent in patients with the presence of lymph node and distant metastases. It is suggested that *TEX14* and ADAM17 may serve as potential biomarkers of CRC progression, influencing the staging, invasion, and metastasis of this malignancy [77]. Interestingly, higher concentration of ADAM10 and ADAM17 proteins in the CRC tissue from the resection margin correlates with higher concentrations of ADAM10 and ADAM17 proteins in the tissue from the tumor, suggesting the presence of shared regulatory mechanisms or a field effect within the tumor microenvironment [78].

Despite the consistent upregulation of ADAM17 in CRC, the available evidence remains heterogeneous. Most studies are based on tumor tissue analyses at the mRNA or protein level, while data on circulating levels are limited, restricting their potential as a non-invasive biomarker. Reported associations with clinicopathological features also vary, with some studies linking ADAM17 to TNM stage and metastasis, whereas others focus primarily on mechanistic aspects, including regulation of the TGF-β/Smad pathway and its effect on cell migration and invasion. In addition, a substantial part of the evidence derives from in vitro and in vivo models with differing experimental conditions, limiting direct comparison with clinical data.

## 6. Multi-Omics Integration

**Figure 3:**
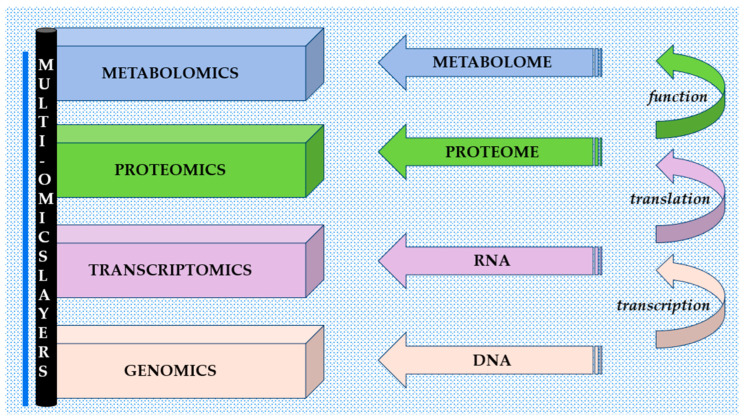
Multi-omics layers.

Recent advances in CRC biomarker research have increasingly shifted toward integrative multi-omics approaches, enabling a more comprehensive characterization of diagnostic and prognostic candidates beyond single-layer expression data. Large-scale in silico analyses using datasets such as The Cancer Genome Atlas (TCGA) and Gene Expression Omnibus (GEO) (e.g., *GSE68468*) have been instrumental in uncovering the complex regulation of these proteins. For instance, studies on ADAM12 integrating transcriptomic data with clinical outcomes and experimental validation have demonstrated that its upregulation is closely associated with EMT signatures and activation of the Wnt/β-catenin signaling pathway. Importantly, the combination of bioinformatics, IHC, and functional assays provides a stronger prognostic value than single-layer analyses [68]. Furthermore, spatial and single-cell approaches have refined our understanding of ADAM-mediated cellular crosstalk within the TME. These analyses reveal that ADAM expression is often compartment-specific. For example, ADAM9 is enriched in cancer CAFs, particularly in tumors with immature desmoplastic reactions. This fibroblast-specific expression promotes tumor progression and migration, emphasizing that the functional landscape of ADAMs extends beyond tumor cell-intrinsic mechanisms and involves stromal–epithelial interactions [65]. In addition, ADAM15 has been implicated in the regulation of immune cell recruitment. Its upregulation correlates with a pro-tumorigenic microenvironment, whereas its loss is associated with increased infiltration of dendritic cells and T lymphocytes [71]. Together, these findings suggest that ADAMs may play a central role in shaping the immune response in CRC. Figure 3 summarizes multi-omics layers [79].

Although fully integrated multi-omics analyses of ADAM family members in CRC remain limited, emerging studies based on TCGA datasets approaches provide important initial insights into their complex regulation.

## 7. Conclusions

CRC remains a major global health challenge, highlighting the urgent need for reliable biomarkers that could improve early detection and prognostic assessment. ADAM proteins, characterized by their unique multi-domain architecture and proteolytic activity, play a fundamental role in the tumorigenesis of CRC. The functional activity of ADAM proteins is strictly controlled by complex regulatory mechanisms, including prodomain cleavage through the “cysteine switch”, conformational activation, and accessory protein-mediated trafficking. TspanC8 and iRhoms are essential for the maturation and proper localization of certain ADAMs like ADAM10 and ADAM17, respectively. Furthermore, their proteolytic capacity is modulated by natural inhibitors such as TIMP3.

A growing body of evidence suggests that among all ADAM family members, especially ADAM8, ADAM9, ADAM12, ADAM15, and ADAM17 have been established to play a role in CRC pathogenesis, which was confirmed by recent original publications. Therefore, this review underscores the potential of selected ADAM proteins as novel biomarkers of CRC progression as well as prognosis and diagnosis of patients with this common malignancy.

Elevated expression of ADAM8 and ADAM12 was observed in CRC tissues and correlated with advanced tumor stage, such as deeper invasion, and the presence of lymph node and distant metastases; increased serum ADAM15 concentrations were linked to the presence of distant metastases, while ADAM9 stimulated CRC cell migration, promoting dissemination, especially in patients with immature-type desmoplastic reaction. Moreover, elevated ADAM9 and ADAM15 expression was associated with poorer survival of CRC patients; likewise, ADAM8 overexpression was proven to be an independent prognostic factor for both OS and DFS, particularly in patients with advanced TNM stages. Similarly, elevated ADAM17 expression was related to the presence of metastasis and poor prognosis of CRC patients. In addition, the measurement of serum ADAM15, especially in combination with CEA, may improve the diagnosis of patients with CRC.

In conclusion, selected ADAM metalloproteinases are critical contributors to the development and progression of CRC, affecting tumor growth, EMT, and metastasis. ADAM8, ADAM9, ADAM12, ADAM15, and ADAM17 were identified as promising biomarkers for the assessment of CRC progression, and were also proven to be prognostic indicators for patients with this malignancy. Moreover, ADAM15 could be a valuable biomarker in the diagnosis of CRC patients, especially in combined measurement with the well-established tumor marker CEA.

Further studies, particularly those focusing on standardized serum-based analyses, are necessary to confirm their usefulness in clinical practice.

## Figures and Tables

**Table 1:** Comparison of selected ADAMs in CRC.

Feature	ADAM8	ADAM9	ADAM12	ADAM15	ADAM17
Expression in CRC tissues	Elevated expression in CRC tissues compared to adjacent non-tumorous samples [62].	Elevated expression in CRC tissues compared to non-tumor samples [64].	Elevated expression in CRC tissues compared to normal mucosa [68].	Elevated expression in colon and rectal tumor tissues compared to adjacent [71].	Elevated expression in primary and metastatic CRC tissues compared to normal mucosa [74].
Serum levels	Limited data	Limited data	Increased serum concentration of ADAM12 in CRC patients compared to healthy controls [69].	Increased serum concentration of ADAM15 in CRC patients compared to healthy controls [70].	Limited data
Clinicopathological correlations	Overexpression associated with advanced tumor stage, lymph node involvement, presence of distant metastasis, and greater tumor invasiveness [63].	Associated with enhanced invasive and migratory potential of CRC cells; CAF-derived ADAM9 linked to tumor progression and desmoplastic reaction [65].	Overexpression correlated with advanced tumor stage, greater depth of invasion, lymph node involvement, the presence of distant metastasis, vascular invasion, and perineural invasion [67].	Elevated serum levels associated with the presence distant metastases [70].	Overexpression associated with advanced TNM stage, poor tumor differentiation, lymph node involvement, and distant metastasis [77].
Clinical outcome	Elevated ADAM8 expression associated with poorer overall survival (OS) and disease-free survival (DFS) [63].	CAF-derived ADAM9 expression associated with poorer survival in CRC patients, particularly in cases with immature desmoplastic reaction [65].	Increased serum concentration of ADAM12 associated with poorer overall survival (OS) [69].	Increased ADAM15 expression associated with poorer overall survival (OS) [71].	Elevated ADAM17 expression associated with poorer prognosis and reduced survival in CRC patients [74].

## Data Availability

No new data were created or analyzed in this study. Data sharing is not applicable to this article.

## Abbreviations

5-FU	5-fluorouracil
ADAM	a disintegrin and metalloproteinase
ADAM9s	secreted ADAM9 isoform
AI	apoptotic index
AJCC	American Joint Committee on Cancer
Akt kinase	serine/threonine kinase
C	cysteine-rich segment
CA 19.9	cancer antigen 19-9
CA 50	cancer antigen 50
CA 72.4	cancer antigen 72.4
CAFs	cancer-associated fibroblasts
CDK	cyclin-dependent kinase
CEA	carcinoembryonic antigen
c-MYC	cellular myelocytomatosis oncogene
RX(R/K)R	arginine (R)/any amino acid (X)/arginine or lysine (R/K)/arginine (R)
CRC	colorectal cancer
D	disintegrin region
DFS	disease-free survival
DNA	deoxyribonucleic acid
DR	desmoplastic reaction
EGFR	epidermal growth factor receptor
EMT	epithelial–mesenchymal transition
ER	endoplasmic reticulum
ERK	extracellular signal-regulated kinase
FAP	familial adenomatous polyposis
FRMD8	FERM Domain-Containing Protein 8
GP	glycoprotein
GSK-3β	glycogen synthase kinase-3 beta
HVR	hypervariable region
IHC	immunohistochemical
iRhoms	rhomboid-like proteins
iTAP	iRhom tail-associated protein
MMP	matrix metalloproteinase
MP	metalloprotease domain
MPD	membrane-proximal domain
mRNA	messenger ribonucleic acid
MSAB	methyl 3-(4-methylphenylsulfonamide)benzoate
mTOR	mechanistic target of rapamycin
OS	overall survival
p38 MAPK	p38 mitogen activated protein kinase
PDI	protein disulfide isomerase
SH3	Src homology 3
Smad	suppressor of mothers against decapentaplegic
TACE	tumor necrosis factor-α-converting enzyme
TEX14	testis-expressed gene 14
TGF	transforming growth factor-α
Thr735	threonine 735
TIMP	tissue inhibitor of metalloproteinase
TM	transmembrane
TME	tumor microenvironment
TMD1	first transmembrane domain
TNF-α	tumor necrosis factor-alpha
TNM	Tumor–Node–Metastasis
Tspans	tetraspanins
Wnt	wingless-related integration site
